# Interleukin-17A is involved in mechanical hyperalgesia but not in the severity of murine antigen-induced arthritis

**DOI:** 10.1038/s41598-017-10509-5

**Published:** 2017-09-04

**Authors:** Matthias Ebbinghaus, Gabriel Natura, Gisela Segond von Banchet, Susanne Hensellek, Martin Böttcher, Birgit Hoffmann, Firas Subhi Salah, Mieczyslaw Gajda, Thomas Kamradt, Hans-Georg Schaible

**Affiliations:** 1 Institute of Physiology 1, University Hospital - Friedrich Schiller University Jena, Teichgraben 8, D-07740 Jena, Germany; 2 Institute of Immunology, University Hospital - Friedrich Schiller University Jena, Leutragraben 3, D-07740 Jena, Germany; 3 Biomolecular Photonics Group, University Hospital - Friedrich Schiller University Jena, Nonnenplan 4, D-07740 Jena, Germany; 4 Institute of Pathology, University Hospital - Friedrich Schiller University Jena, Ziegelmühlenweg 1, D-07740 Jena, Germany; 5 Iraqi centre for cancer and medical genetics research, Almustansiriyah University, Baghdad, Iraq; 6 Present Address: Department of Internal Medicine 5 - Hematology and Oncology, University Hospital of Erlangen, Schwabachanlage 12, D-91054 Erlangen, Germany

## Abstract

Interleukin-17A (IL-17A) is considered an important pro-inflammatory cytokine but its importance in joint diseases such as rheumatoid arthritis (RA) is unclear. It has also been reported that IL-17A may induce pain but it is unclear whether pro-inflammatory and pro-nociceptive effects are linked. Here we studied in wild type (WT) and IL-17A knockout (IL-17AKO) mice inflammation and hyperalgesia in antigen-induced arthritis (AIA). We found that the severity and time course of AIA were indistinguishable in WT and IL-17AKO mice. Furthermore, the reduction of inflammation by sympathectomy, usually observed in WT mice, was preserved in IL-17AKO mice. Both findings suggest that IL-17A is redundant in AIA pathology. However, in the course of AIA IL-17AKO mice showed less mechanical hyperalgesia than WT mice indicating that IL-17A contributes to pain even if it is not crucial for arthritis pathology. In support for a role of IL-17A and other members of the IL-17 family in the generation of pain we found that sensory neurones in the dorsal root ganglia (DRG) express all IL-17 receptor subtypes. Furthermore, in isolated DRG neurones most IL-17 isoforms increased tetrodotoxin- (TTX-) resistant sodium currents which indicate a role of IL-17 members in inflammation-evoked sensitization of sensory nociceptive neurones.

## Introduction

Interleukin-17A (IL-17A), before 2003 in the context of autoimmune diseases commonly only called IL-17, and five additional cytokines (IL-17B-F) form a family of closely related cytokines^1, 2^. IL-17A, the prototype member of the IL-17 family^3^, is secreted from Th17, CD8^+^T, γ∂T, natural killer cells, activated monocytes and neutrophils^3^. As a mediator of both innate and adaptive immunity^2, 4, 5^, IL-17 is involved in the defence against bacteria and fungi^6, 7^ as well as in the pathogenesis of autoimmune diseases such as rheumatoid arthritis (RA), multiple sclerosis, and others^2–5^. Drugs targeting “IL-17 signalling” are in preclinical development and clinical trials for the treatment of chronic inflammatory disorders^8^. However, particularly in RA, the role of IL-17 is not well understood. The inflamed synovium of RA patients contains plenty of Th17 cells and the IL-17 levels in the synovial fluid correlate with disease severity^9, 10^. IL-17A induces the release of chemokines and cytokines (IL-1β, IL-6, IL-8, RANKL, TNF), matrix metalloproteinases, nitric oxide and prostaglandin E_2_ from fibroblasts, osteoblasts, chondrocytes and macrophages, all cells involved in RA pathogenesis^9, 11–14^. However, s*ecukinumab*, an antibody against IL-17A, is only approved for the treatment of psoriasis arthritis and ankylosing spondylitis while IL-17A neutralisation in RA had less convincing clinical effects so far^15^.

Interestingly, in experimental mouse models of arthritis the pathogenic role of IL-17 is also heterogeneous. While collagen-induced arthritis (CIA) depends on the presence of IL-17^16^, K/BxN serum-transfer arthritis^17^, proteoglycan-induced arthritis (PGIA)^18^ and staphylococcus aureus-induced arthritis^19^ seem to depend less on IL-17 signalling. In the model of antigen-induced arthritis (AIA), IL-17A knock-out (IL-17AKO) mice showed reduced swelling at the very beginning^20^. The severity of AIA in wild type (WT) mice was only weakly attenuated by blocking IL-17^21^.

In addition to its role in inflammatory processes, IL-17A may play a role in the generation of inflammatory pain, as shown in behavioural experiments in AIA^21, 22^ and in acute inflammatory conditions^23–25^. Several findings suggest that IL-17A can act on nociceptive sensory neurones. Immunohistochemically, a large proportion of sensory neurones express IL-17A receptors, and bath application of IL-17A generated hyperexcitability in small to medium-sized isolated and cultured dorsal root ganglion (DRG) neurones^21^. The injection of IL-17A into a normal knee joint increased the responses of nociceptive C-fibres to mechanical stimuli to the knee joint^21^. Because pain is a major symptom of arthritis, the neuronal effects of IL-17 deserve further attention.

A further observation on IL-17A was made in the context of sympathectomy. In several arthritis models sympathectomy significantly attenuated the severity of inflammation^26, 27^. We found previously that in the acute stage of murine AIA chemical sympathectomy strongly reduced the severity of inflammation and hyperalgesia^28^. Sympathectomised C57BL/6 WT mice showed decreased amounts of IL-2 and a particularly striking reduction of IL-17A produced by lymphocytes *in vitro* ^28^ raising the hypothesis that the anti-inflammatory effect of sympathectomy depends mechanistically on IL-17A.

The present study had three major aims. First, we aimed to determine if a putative role of IL-17A in the generation of pain is linked to a pathogenic role in arthritis. Using WT and IL-17AKO mice we explored whether IL-17A is critically involved in the development of both arthritis and arthritis-evoked pain. Second, in order to test the hypothesis that the anti-inflammatory effect of sympathectomy critically depends on the reduction of IL-17A signalling we investigated whether the effect of sympathectomy on AIA severity is abolished or maintained in IL-17AKO mice. Third, in order to generate mechanistic insights into the role of IL-17A and other members of the IL-17 family in nociception, we explored the expression of IL-17 receptors in the dorsal root ganglia, and we tested whether Na^+^ currents of isolated cultured sensory neurones are similarly influenced by IL-17A and other IL-17 family members.

## Results

### Severity of AIA and hyperalgesia in WT and IL-17AKO mice

**Figure 1:**
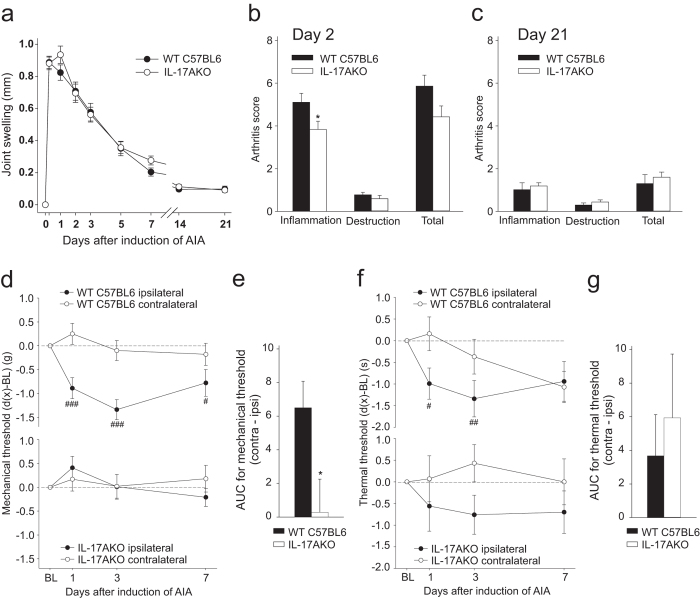
Joint swelling, histopathological scoring and pain-related behaviour in wild type (WT) mice and IL-17AKO mice in AIA. (**a**) No significant differences in joint swelling at the injected knee were detectable in comparison of multiple experiments of WT mice and IL-17AKO mice in the course of AIA (n = 31–63 per group). (**b**,**c**) Histological examination of the inflamed knee joints from WT and IL-17AKO mice at day 2 (n = 6–23 per group) and day 21 of AIA (n = 12–16 per group) showed no significant differences in total arthritis scores between both groups of mice. (**d**) Hashes indicate a reduction of mechanical withdrawal threshold at the paw ipsilateral to the inflamed knee (black symbols) in WT mice (top) (n = 43). IL-17AKO mice did not show a drop of the mechanical threshold (bottom) (n = 27). (**e**) Areas between the ipsilateral and contralateral curves in (**d**) indicating the magnitude of mechanical hyperalgesia. (**f**) A significant reduction of thermal withdrawal threshold at the ipsilateral paw was seen in WT mice (top) (n = 34–43) whereas IL-17AKO mice (bottom) showed only a tendency of reduction of threshold (n = 27). (**g**) AUC from (**f**) indicating no significant differences in the magnitude of thermal hyperalgesia between both groups of mice. Values are mean ± SEM. *p < 0.05 (two-tailed Student’s t-test), ^#^p < 0.05, ^##^p < 0.01 (Wilcoxon’s matched-pairs signed-rank test). *AIA* antigen-induced arthritis, *AUC* area under the curve, *BL* baseline.

In order to explore whether IL-17A is essential for a full expression of murine AIA, we compared the course and severity of arthritis in WT and IL-17AKO mice. The injection of mBSA into the right knee joint of immunized animals caused a rapidly developing swelling of the joint with a peak at 1–2 days after AIA induction. The severity and time course of joint swelling in WT and IL-17AKO mice were almost overlapping (Fig. 1a). Compared to WT mice IL-17AKO mice showed a slightly reduced (p = 0.047) histological synovitis score at day 2 after injection. However, the total overall arthritis scores did not significantly differ at days 2 and 21 (Fig. 1b,c) after induction of AIA.

Nevertheless, WT and IL-17AKO mice showed considerable differences in the pain-related behaviour during AIA. While both WT and IL-17AKO mice showed guarding of the ipsilateral leg, only WT mice exhibited secondary mechanical hyperalgesia at the ipsilateral paw. WT mice showed a significant drop of the mechanical threshold at the ipsilateral paw in acute AIA (Fig. 1d, top; hashes indicate significant differences of thresholds compared to baseline values; d1 p < 0.001, d3 p < 0.001, d7 p = 0.015) indicating secondary mechanical hyperalgesia at the ipsilateral paw. By contrast, IL-17AKO mice did not exhibit such a reduction of threshold (Fig. 1d, bottom). Figure 1e shows the significant difference (p = 0.017) of the magnitude of secondary mechanical hyperalgesia, calculated as area under the curve (AUC) for the values of the ipsi- and contralateral paw displayed in Fig. 1d. WT mice also showed a significant drop of the thermal threshold at the ipsilateral paw in acute AIA (Fig. 1f, top, d1 p = 0.014, d3 p = 0.005) whereas IL-17AKO mice exhibited only a tendency (Fig. 1f, bottom). The left-right difference of thermal threshold was similar in WT and IL-17AKO mice, and the AUCs did not indicate a significant difference of thermal hyperalgesia in WT and IL-17AKO mice (Fig. 1g). Thus deletion of IL-17A significantly reduces inflammatory secondary mechanical hyperalgesia in AIA although it does not decrease AIA severity.

### Effect of sympathectomy on the severity of AIA in IL-17AKO mice

**Figure 2:**
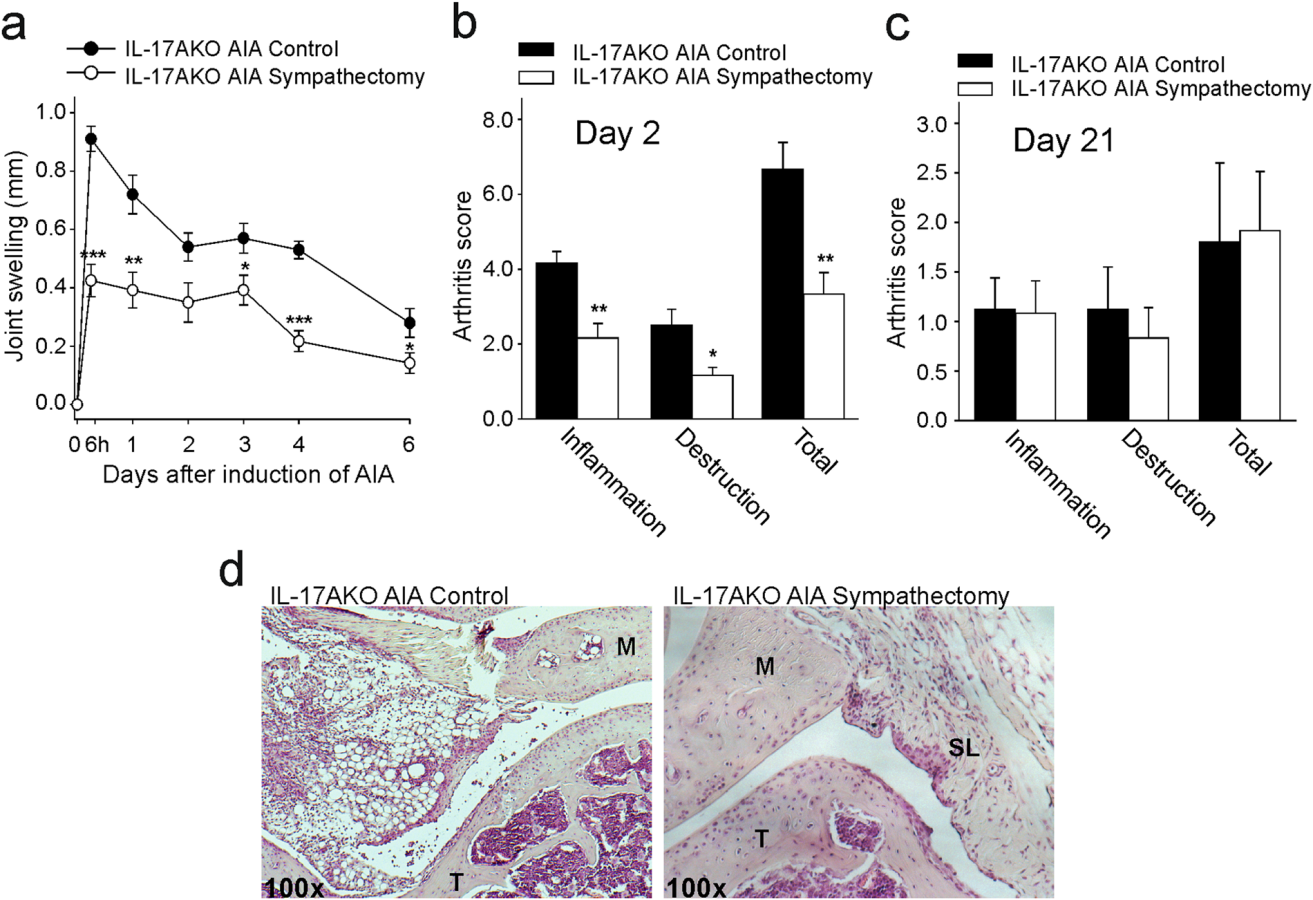
Joint swelling and histopathological scores in AIA in IL-17AKO mice after sympathectomy. (**a**) Reduction of joint swelling by sympathectomy (n = 6 per group). (**b**) Reduction of arthritis score in acute AIA after sympathectomy (n = 6 per group). (**c**) At day 21 of AIA the differences between sympathectomised and non-sympathectomised mice disappeared (n = 6 per group). (**d**) Typical histology after H&E staining at day 2 of AIA from IL-17AKO mice. AIA controls (left side) show stronger signs of inflammation (infiltration of immune cells, hyperplasia of synovial lining cells and surface defects of cartilage and bone) than sympathectomised mice. M, meniscus; T, tibia; SL, synovial layer. Magnification 100x. Values are mean ± SEM. *p < 0.05, **p < 0.01, ***p < 0.001 (two-tailed Student’s t-test).

In order to investigate whether the anti-inflammatory effect of sympathectomy critically depends on the reduction of IL-17A signalling, we studied the effect of sympathectomy in IL-17AKO mice. Sympathectomy did not alter the incidence of AIA in IL-17AKO mice which remained 100%. However, sympathectomy at day 1 before AIA induction reduced the severity of AIA. In sympathectomised mice the joint swelling was significantly reduced (Fig. 2a; 6h p < 0.001, d1 p = 0.005, d3 p = 0.037, d4 p < 0.001, d6 p = 0.043), and the histological arthritis score at day 2 of AIA was also significantly decreased (Fig. 2b; inflammation p = 0.002, destruction p = 0.019, total p = 0.005). However, in later stages of AIA (from day 7 on) the effect of sympathectomy was no longer visible (Fig. 2c). Representative histological specimens of inflamed joints (day 2 of AIA), either from AIA control animals or from AIA animals after sympathectomy, are shown in Fig. 2d.

Essentially, the anti-inflammatory effect of sympathectomy in IL-17AKO mice was similar to that observed previously in WT mice^28^. The Supplementary Figure S1 shows the reduction of swelling and total arthritis score by sympathectomy in IL-17AKO mice and WT mice side by side. One day after induction of AIA the maximum reduction of swelling was ~46% in IL-17AKO mice and ~42% in WT mice.

**Figure 3:**
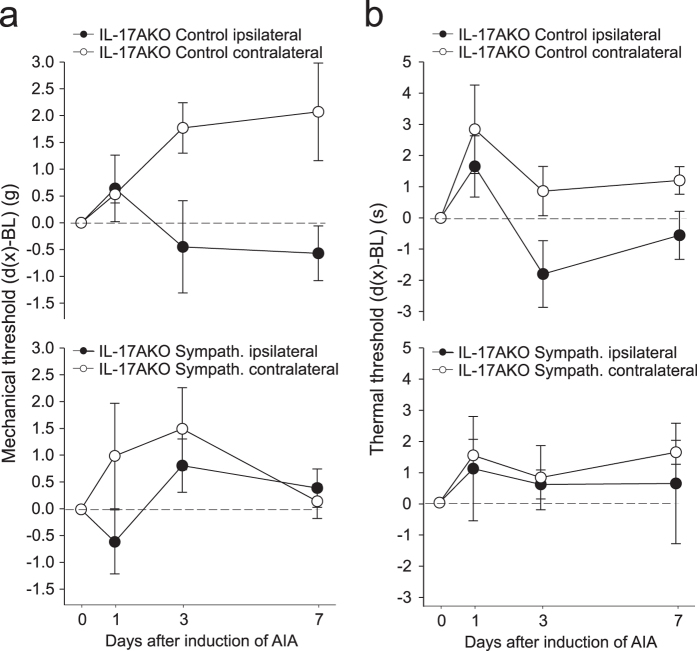
Testing of mechanical and thermal hyperalgesia in non-sympathectomised and sympathectomised IL-17AKO mice before and during the first 7 days of AIA (in each group n = 4). (**a**) No consistent reduction of mechanical threshold during AIA in the ipsilateral paw. Rather an increase of threshold on the contralateral side. (**b**) No consistent effects regarding thermal threshold.

Considering the effect of sympathectomy on pain it is important to note that IL-17AKO mice did not show secondary mechanical hyperalgesia during AIA. Hence an effect of sympathectomy on pain behaviour could not be expected. We tested 4 IL-17AKO mice with sympathectomy and 4 IL-17AKO mice without sympathectomy for responses to mechanical and stimuli in the first 7 days of AIA. Neither control mice with AIA (Fig. 3a, top) nor sympathectomised mice with AIA (Fig. 3a, bottom) showed a consistent reduction of the threshold at the ipsilateral during AIA (black dots in Fig. 3a), consistent with the data shown in Fig. 1d, bottom. On the contralateral side (circles) the mechanical threshold was even slightly increased. Concerning thermal threshold non-sympathectomised IL-17AKO mice showed a similar tendency as displayed in Fig. 1f bottom, and after sympathectomy no effect on thermal threshold was observed (Fig. 3b). In sum, sympathectomy acts anti-inflammatory in both WT and IL-17AKO mice but has an anti-nociceptive effect only in WT mice.

### Cellular and humoral immune responses and levels of CGRP in acute AIA in IL-17AKO mice after sympathectomy

**Figure 4:**
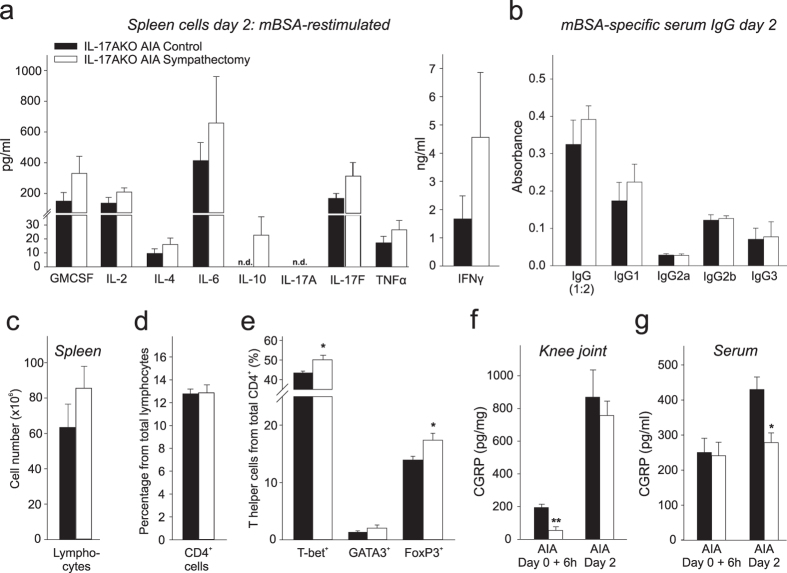
Immune profile and levels of CGRP in IL-17AKO mice in acute AIA. (**a**) Cytokine levels in supernatants of *in vitro* antigen (mBSA)-specifically re-stimulated spleen cells isolated on day 2 of AIA (n = 6 per group). (**b**) Serum levels of antigen-specific immunoglobulins on day 2 of AIA (n = 6 per group). Specification in brackets defines the dilution of the immunoglobulin G value. No significant differences were seen between sympathectomised and control AIA mice, neither for cytokines nor for immunoglobulin levels. (**c**–**e**) Flow cytometry from single cell suspensions from spleens from IL-17AKO mice, removed at day 2 of AIA (n = 5 per group). Fractions of total single lymphocytes (**c**), CD4^+^ cells (**d**) and percentages of T helper 1 (T-bet^+^), T helper 2 (GATA3^+^) and T regulatory cells (FoxP3^+^) (**e**). Sympathectomy slightly increases the percentages of T-bet^+^ and FoxP3^+^ splenic lymphocytes. (**f**) Levels of CGRP tissue content in total knee joint extracts of IL-17AKO mice 6 h and 2 days after induction of AIA in relation to total protein concentration (n = 5–6 per group). (**g**) Levels of CGRP in serum of IL-17AKO mice at time points according to (**f**) (n = 5–6 per group). Sympathectomised IL-17AKO mice show significantly less CGRP in knee joints 6 h after induction of AIA and in serum 2 days after induction of AIA. Values are mean ± SEM. n.d., not detectable. *p < 0.05, **p < 0.01 *AIA* antigen-induced arthritis, *CGRP* calcitonin gene-related peptide, *Ig* immunoglobulin, *mBSA* methylated bovine serum albumin.

In WT mice we had shown before that the cytokine release from lymphocytes is altered by sympathectomy^28^. In order to find mechanisms which explain the anti-inflammatory effect of sympathectomy in IL-17AKO mice, we compared materials from acute AIA control mice and from mice which received sympathectomy before AIA induction. We used spleens as source of lymphocytes because spleens are adrenergically innervated and because sympathectomy in WT mice induced the same effects on cytokine release in splenic and lymph node-derived lymphocytes^28^. The release of cytokines was not significantly different from mBSA-restimulated spleen cells of control and sympathectomised mice (Fig. 4a). However, in particular the release of IL-10 and interferon-γ was higher in spleen cells from sympathectomised mice although the difference to spleen cells from AIA control mice was not significant. Notably, we could also detect IL-17F produced by lymphocytes of IL-17AKO mice. No significant differences were found for antigen-specific IgG in the serum (Fig. 4b). The discrete changes of cytokine release were accompanied by changes in the splenic lymphocyte population (Fig. 4c–e). While the fraction of CD4^+^ cells from total lymphocytes was not altered by sympathectomy the percentages of T helper 1 (T-bet^+^) and T regulatory (FoxP3^+^) cells from CD4^+^ cells were slightly increased (T-bet^+^ p = 0.029, FoxP3^+^ p = 0.034) after sympathectomy. T helper 1 and T regulatory cells are sources of interferon-γ and IL-10 respectively^29^.

We also measured the concentration of the neuropeptide CGRP in the joint and in the serum. CGRP is released from sensory neurones and produces neurogenic inflammation^30^. The tissue content of CGRP in the knee joint was significantly (p < 0.001) reduced at 6 hours after induction of AIA but not at day 2 of AIA (Fig. 4f), whereas the serum concentration of CGRP was significantly (p = 0.01) smaller in sympathectomised mice on day 2 of AIA (Fig. 4g). Thus sympathectomy in IL-17AKO mice has only a minor influence on the release of cytokines and immunoglobulins but the concentration of CGRP is significantly decreased in joints and serum in acute AIA.

### Expression of IL-17 receptors in dorsal root ganglia

**Figure 5:**
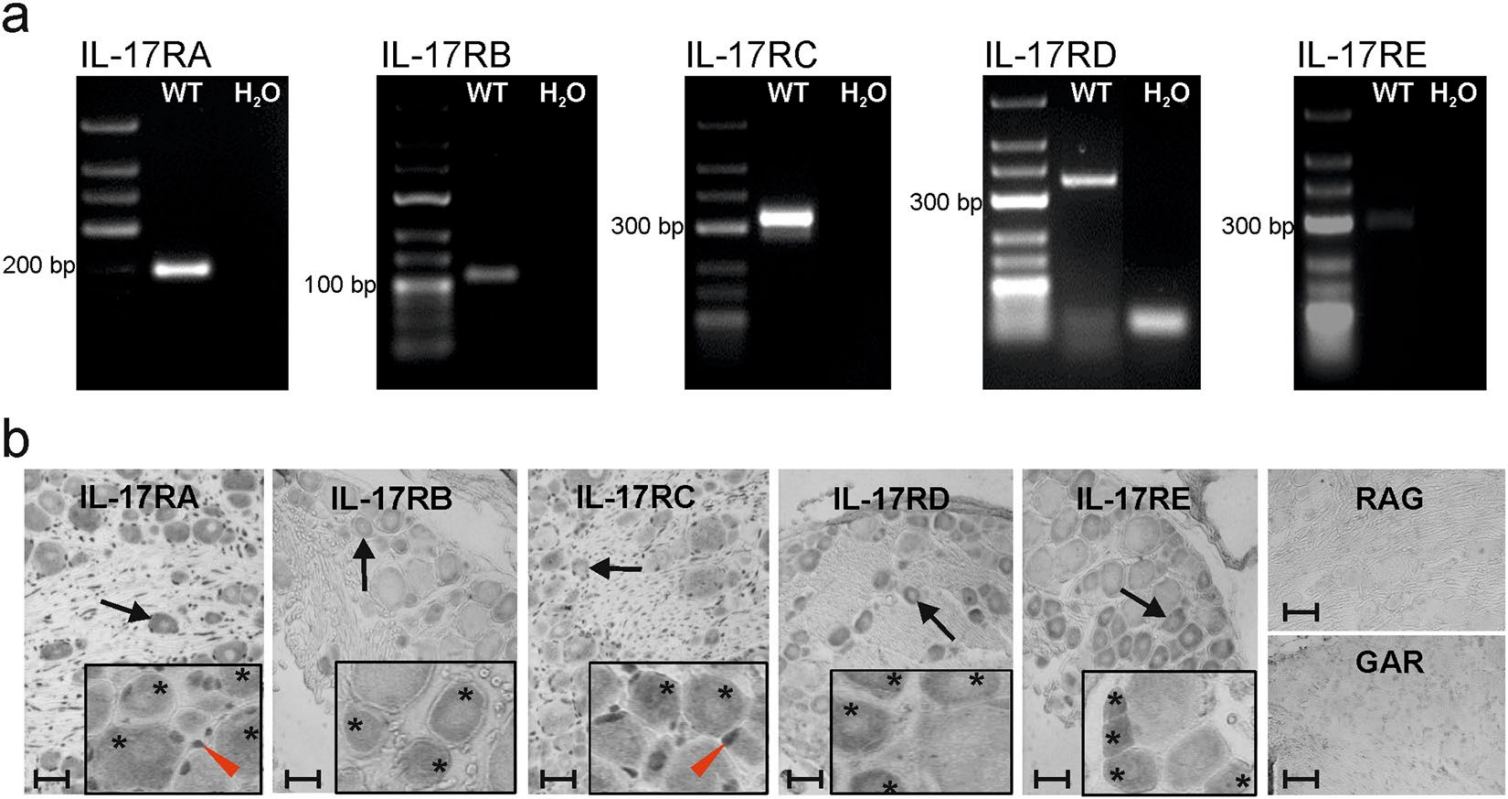
Expression of IL-17 receptors in DRG neurones in WT mice. (**a**) Representative agarose gel images of PCR products of IL-17 receptor subtypes in dorsal root ganglion (DRG) neurones of naïve WT mice. In WT mice a strong expression of IL-17RA, IL-17RC and IL-17RD and a weak expression of IL-17RB and IL-17RE is detectable. (**b**) Typical labelling of IL-17 receptors in DRG sections from naïve WT mice with anti-IL-17RA, -IL-17RB, -IL-17RC, -IL-17RD and anti-IL17RE antibodies. Arrows indicate representative cells considered positively labelled. Insets display labelling of sections at higher magnification and show that all the receptor subtypes were localised in subsets of neurones of different sizes (asterisks indicate all cells considered positively labelled) whereas IL-17RA and IL-17RC were also localised in satellite cells (exemplary cells marked by red triangles). Control staining only with rabbit-anti-goat biotinylated antibody (RAG) and goat-anti-rabbit biotinylated antibody (GAR). Magnification 200x (bars 15 µm), insets 400x. Values are mean ± SEM. *IL-17R* IL-17 receptor, *H*_2_0, water control, *bp* base pairs.

The reduction of mechanical hyperalgesia in spite of the unaltered severity and course of AIA in IL-17AKO mice suggests that IL-17A acts on sensory neurones. While we previously showed the expression of IL-17 receptor (R)A in DRG neurones^21^ it was unknown whether other IL-17 receptor subtypes are also expressed in the DRG. Using PCR we found PCR products of IL-17RA, IL-17RB, IL-17RC, IL-17RD and IL-17RE in DRG tissue (Fig. 5a). Using immunohistochemistry we could localise all of the receptor subtypes in subsets of neurones of different sizes in DRG sections (Fig. 5b, all panels display sections at low magnification and insets with higher magnification). In addition, IL17RA and IL-17RC were also localised in satellite cells (Fig. 5b). The panels on the right show control sections in which no staining was observed after omission of the primary antibodies. The expression of all IL-17 receptors in DRG tissue of WT mice strongly suggests that IL-17 acts functionally on nociceptive neurones.

### Expression of other cytokines of the IL-17 family in knee joint tissue and spleen

**Figure 6:**
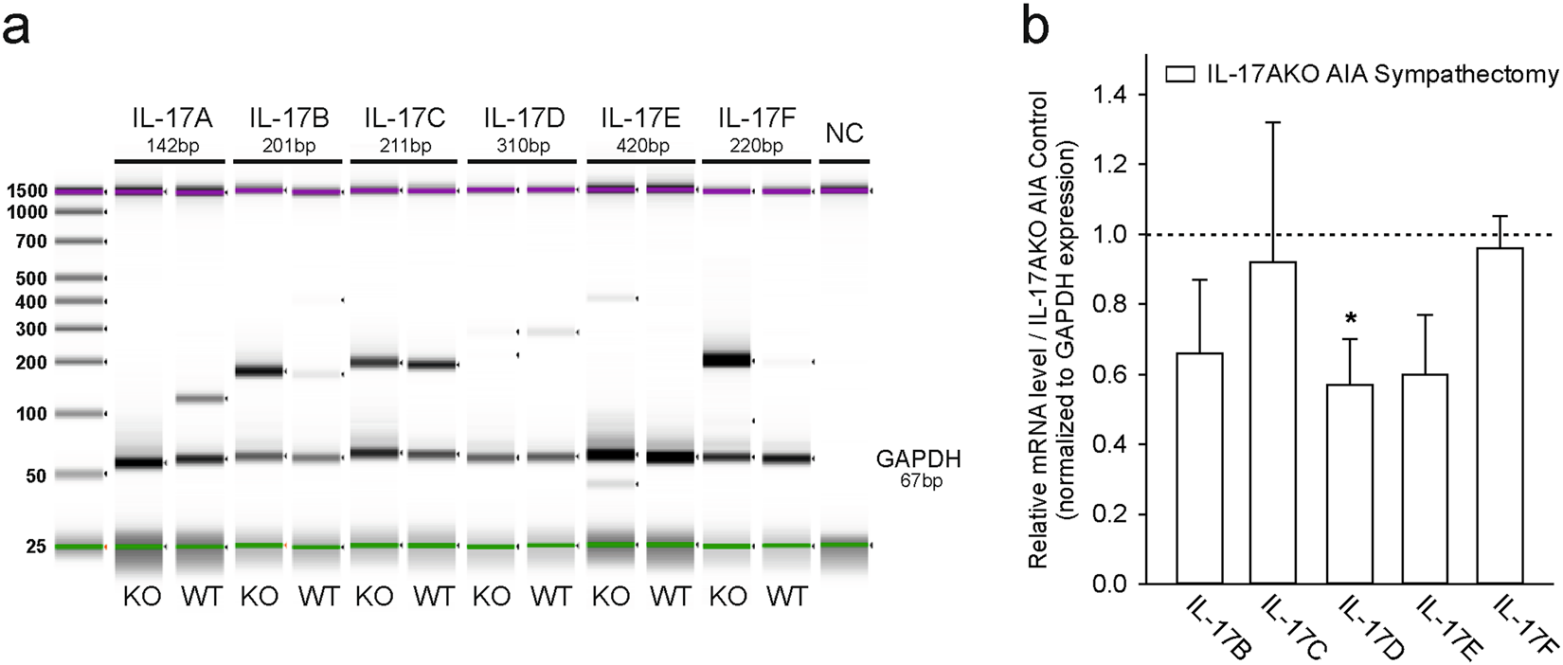
PCR and RT-PCR of IL-17 cytokine family members in knee joint tissues at day 2 of AIA. (**a**) Representative PCR-ScreenTape (Agilent 2200 TapeStation) of IL-17 cytokine family members in tissues of WT and IL-17AKO mice. PCR analysis does not give information about expression quantity. Upper (magenta) and lower (green) markers are used as internal references to determine the molecular weight size of the sample. DNA ladder (25–1500 bp) on the left. GAPDH (Glyceraldehyde 3-phosphate dehydrogenase) serves as a housekeeping control gene. (**b**) Quantitative reverse transcriptase (RT)-PCR in tissues of sympathectomised IL-17AKO mice and IL-17AKO AIA control mice (n = 5 per group). mRNA level after sympathectomy are given in relation to non-sympathectomised control mice. Sympathectomy significantly (p = 0.012) decreases IL-17D-mRNA. Values are mean ± SEM. *p < 0.05 bp, base pairs; NC, no amplification control.

In order to get a more complete picture of the presence of IL-17 family members, we looked for the other IL-17 family members in the knee joint and the spleen. Knee joint tissue extracts of WT mice of day 2 of acute AIA expressed IL-17A, IL-17B, IL-17C, IL-17D and IL-17F, and all of them except IL-17A were also expressed in IL-17AKO mice in various intensities. IL-17E was only visible in knee joint tissue of IL-17AKO mice (Fig. 6a). All IL-17 cytokines were also expressed in the spleen from immunized mice except IL-17A in the spleen of IL-17AKO mice (Supplementary Figure S2). In order to determine if sympathectomy alters the expression of IL-17 cytokines in IL-17AKO mice at the site of inflammation, quantitative reverse transcriptase (RT)-PCR was performed in knee joint tissues from day 2 of AIA. mRNA levels of IL-17B, IL-17D and IL-17E were slightly reduced by sympathectomy in comparison to IL-17AKO AIA control mice (Fig. 6b). IL-17C and IL-17F were not altered. In summary, except IL-17A we detected in IL-17AKO mice all cytokines of the IL-17 family in spleen and knee joint, and sympathectomy partially decreased their expression in joints.

### Effects of IL-17 on Na^+^ currents in DRG neurones and on the release of CGRP

**Figure 7:**
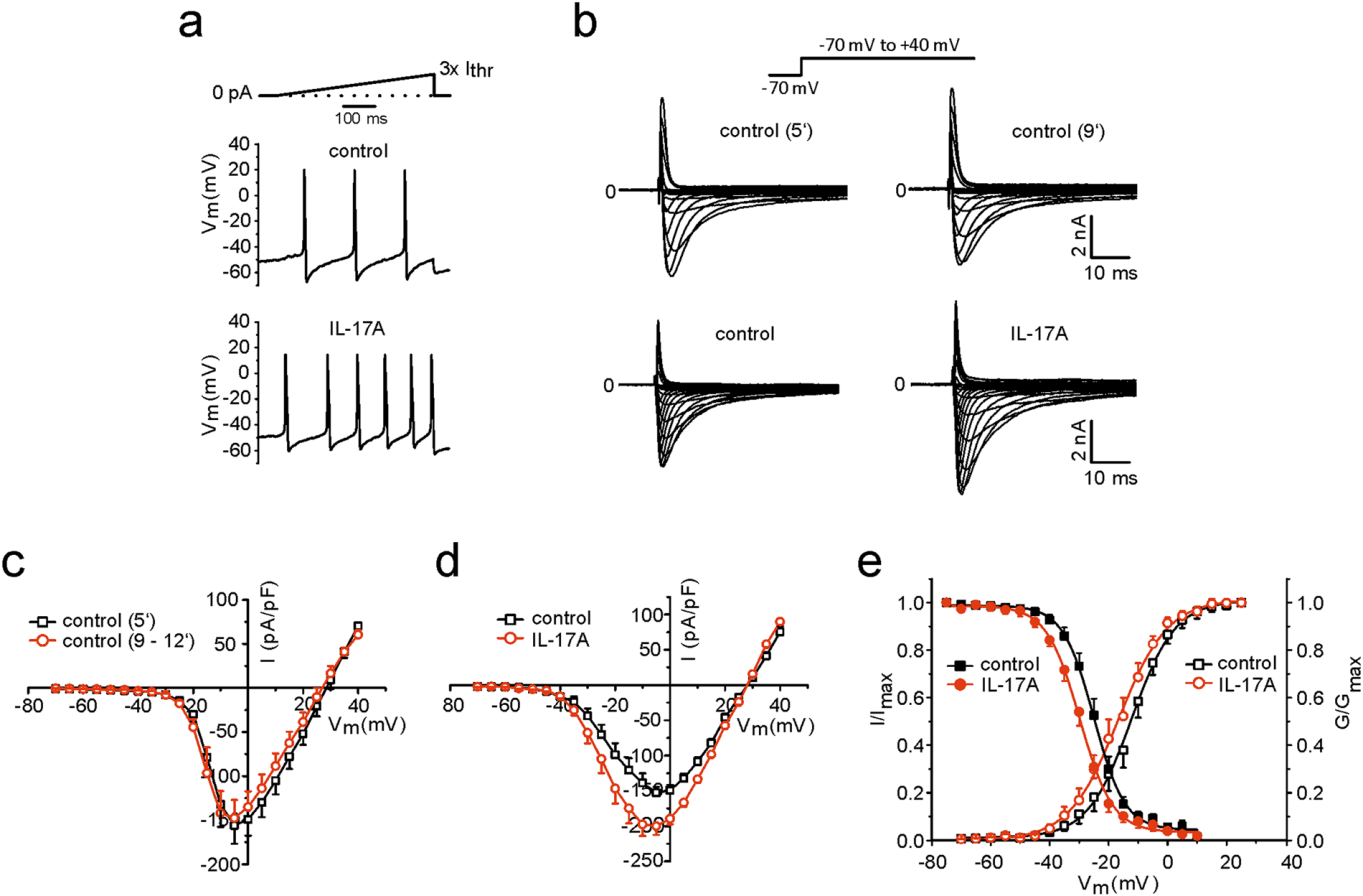
Effects of IL-17A on excitability and on TTX-resistant and TTX-sensitive Na^+^ currents in isolated and cultured DRG neurones. (**a**) Testing of the excitability of a neurone using a ramp-shaped current injection protocol. After application of IL-17A the first action potential appeared at a shorter latency (indicating that less current was necessary) and more action potentials were elicited during the ramp. (**b**) top: voltage-clamp recording from a control DRG neurone showing no increase of TTX-R Na^+^ currents between 5 min (5′, top, left) and 9 min (9′, top, right), bottom: voltage-clamp recording from another neurone showing an increase of TTX-R Na^+^ currents at 5 min after bath application of IL-17A. (**c**) I/V curve of control neurones (n = 10) at 5 min and at 9–12 min of recording. (**d**) I/V curve of control neurones (n = 11) before (5 min) and 5–7 min after bath application of IL-17A. (**e**) Normalised peak conductance (G/G_max_, open symbols) and steady-state inactivation (I/I_max_, filled symbols) before (5 min) and 5–7 min after bath application of IL-17A.

In order to get functional evidence for a role of IL-17 family members in nociception we performed patch-clamp recordings from small- to medium-sized isolated and cultured DRG neurones. Similar as previously shown in isolated rat DRG neurones^21^, IL-17A enhanced the excitability of mouse DRG neurones. Figure 7a shows a current-clamp experiment in which we monitored the occurrence of action potentials during a ramp-shaped current injection before and after IL-17A. After IL-17A the first action potential appeared at a shorter latency (meaning lower current), and more action potentials were elicited. In total 6 of 7 neurones showed a reduction of the current threshold (by 50–100 pA in the step protocol), and 2 of these 6 neurones showed more action potentials in the ramp protocol.

In the further experiments we used voltage-clamp recordings and monitored as readout parameter tetrodotoxin-resistant (TTX-R) and tetrodotoxin-sensitive (TTX-S) Na^+^ currents because Na^+^ currents are modified by numerous mediators which cause nociception and pain. The effect of IL-17A on TTX-R Na^+^ currents are shown in Fig. 7b–e. In control neurones the testing procedure without application of IL-17A did not increase TTX-R Na^+^ currents (see specimen in Fig. 7b, top). Figure 7c displays the I/V curve of 10 neurones. The average peak current density (I_max_) was −161.3 ± 21.9 pA/pF at the beginning of recording and −153.1 ± 23.4 pA/pF (p > 0.05, t-test) after a recording time of 9–12 min, thus showing a typical weak decrease (about 10%) of the current amplitudes (run down) during the recording. The values of V_1/2_ were −12.0 ± 0.1 mV at the beginning and −13.6 ± 0.1 mV at 9–12 min, and the respective slopes were k = 4.2 ± 0.1 at the beginning and k = 4.4 ± 0.1 after 9–12 min. Figure 7b, bottom displays a neurone in which IL-17A caused an increase of the current amplitude. The I/V curve of 11 neurones shows an average increase of the maximal TTX-R Na^+^ current (I_max_) from −166.4 ± 16.2 pA/pF before IL-17A to −213.5 ± 22.7 pA/pF 5 min after bath application of IL-17A, and thus I_max_ increased by 20% (Fig. 7d, p = 0.001; t-test). The normalised peak conductance showed a leftward shift after application of IL-17A (Fig. 7e, open symbols). V_1/2_ changed from −12.2 ± 0.3 mV to −16.7 ± 0.3 mV (p = 0.02, t-test), the slopes from k = 7.4 ± 0.3 to k = 8.4 ± 0.3 mV.

In 7 neurones we tested the steady-state inactivation before and after bath application of IL-17A. The steady-state inactivation curves (Fig. 7e, filled symbols) show a significant shift to negative voltages (p < 0.05, t-test). In the presence of IL-17A half-maximum voltages of the normalized currents moved from −25.0 ± 0.2 to −29.8 ± 0.3 mV at about 5 min after application of IL-17A. The corresponding slopes changed from 5.3 ± 0.2 mV (control) to 5.6 ± 0.2 mV. In the combined plot of normalized activation and inactivation curves in Fig. 7e the window current is visible, i.e. the voltage region where a sustained sodium current is observed. The maximum window currents were found at −19.2 mV (control) and −24.2 mV after IL-17A.

**Figure 8:**
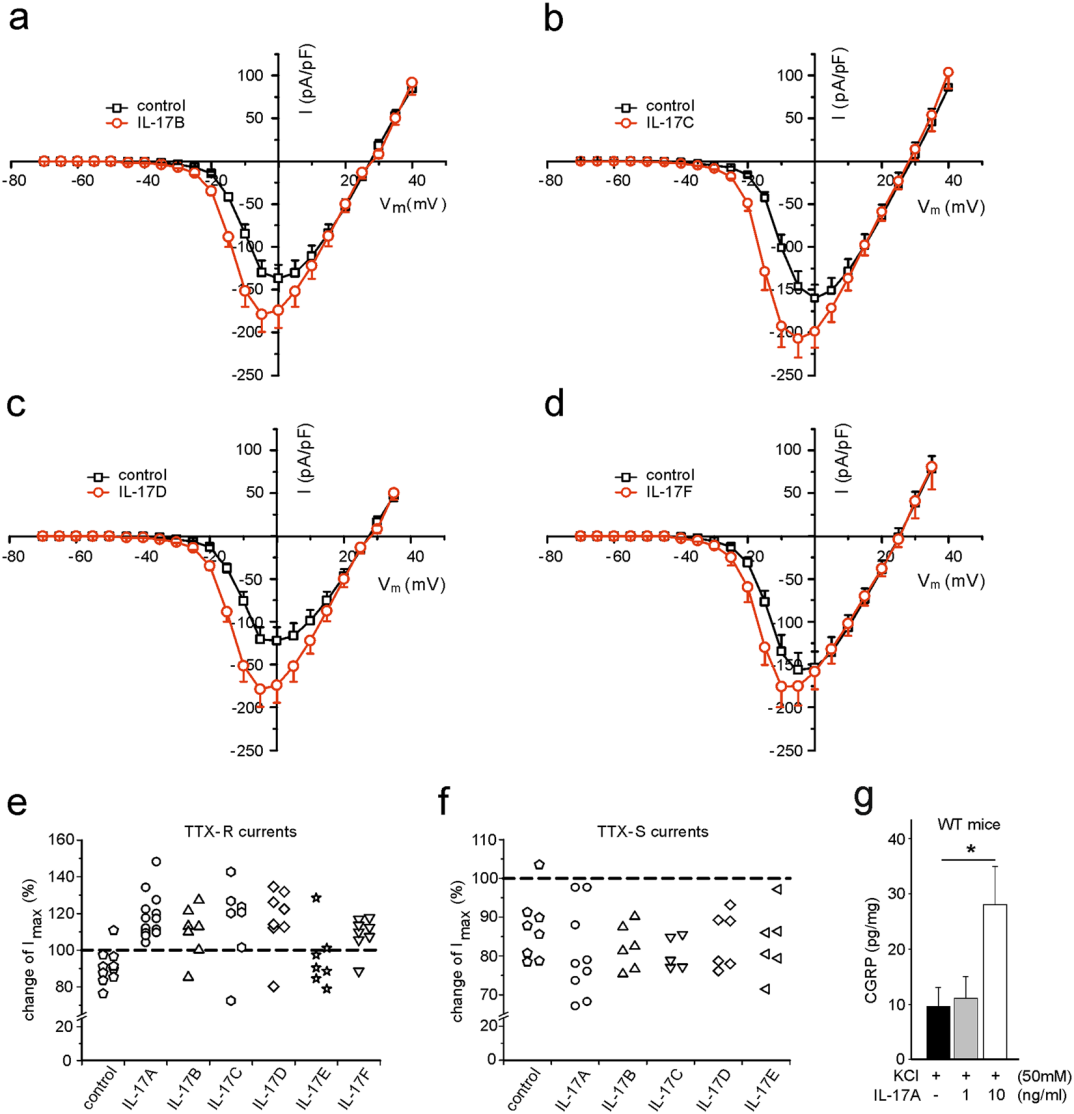
Effects of IL-17 family members on TTX-resistant and TTX-sensitive Na^+^ currents in isolated and cultured DRG neurones and CGRP release *in vitro* from DRG neurones from naïve WT mice. (**a**–**d**) Changes of I_max_ (peak current densities) in DRG neurones after bath application of IL-17B (n = 5) (**a**), IL-17C (n = 5) (**b**), IL-17D (n = 7) (**c**), and IL-17F (n = 7) (**d**). Only the neurones were included which showed increases of TTX-R currents, as indicated in (**e**). (**e**) I_max_ of TTX-R currents of all neurones tested with IL-17 family members. No compound was applied to control neurones. (**f**) Changes of TTX-S Na^+^ currents (I_max_) in DRG neurones after bath application of IL-17 family members. No compound was applied to control neurones. (**g**) Release of CGRP from DRG neurones *in vitro* after cell culture for 48 h and depolarization with KCl and stimulation with IL-17A for 20 min (n = 5 per group). Illustrated CGRP concentrations show the evoked release minus the basal production in the same time in relation to total protein concentration of the DRG cell cultures. Although no significant differences in the release of CGRP between WT and IL-17KO were detectable, IL-17A (10 ng/ml) signalling increases CGRP release in WT DRG neurones (p = 0.045). *p < 0.05 *CGRP* calcitonin gene-related peptide.

Figure 8a–d shows changes of TTX-R Na^+^ currents evoked by IL-17B, IL-17C, IL-17D, and IL-17F, and Fig. 8e summarises the effects of all IL-17 family members on TTX-R Na^+^ currents in all neurones tested. While all except one control neurones showed a small decrease of I_max_ during the recording, the bath application of IL-17A caused an increase of I_max_ in the vast majority of neurones (Fig. 8e). All IL-17 family members except IL-17E increased I_max_ at least in a proportion of neurones (Fig. 8e). Because not all neurones showed an increase of I_max_ after IL-17B, IL-17C, IL-17D, IL-17F, we included in the I/V curves only the neurones with an increase of I_max_. By contrast, during the recording of TTX-S Na^+^ currents in DRG neurones from Na_V_1.8^−/−^ mice rather a decrease was found after application of IL-17A and the other IL-17 family members (Fig. 8f). This decrease was similar as in control neurones, and thus we could not identify an effect of IL-17 isoforms on TTX-S Na^+^ currents. Taken together, these data show that all IL-17 isoforms except IL-17E have the potential to increase the excitability of small- to medium-sized DRG neurones.

In further experiments we explored whether the bath application of IL-17A can activate cultured neurones such that they release CGRP. Using a typical protocol we tested whether IL-17A enhances the release of CGRP evoked by application of KCl. Figure 8g displays the release of CGRP by 50 mM KCl, and it shows an increase of the KCl-evoked CGRP release by 10 ng/ml IL-17A but not by 1 ng/ml IL-17A.

## Discussion

This study provided the following results. First, the severity and time course of AIA was almost indistinguishable in WT and IL-17AKO mice. Nevertheless, IL-17AKO mice showed significantly less secondary mechanical hyperalgesia than WT mice. Second, the reduction of the severity of AIA by sympathectomy was preserved in IL-17AKO mice. Sympathectomy in IL-17AKO mice caused a slight shift towards T helper 1 (T-bet^+^) and T regulatory (FoxP3^+^) cell fractions, and it reduced the release of CGRP in the acute phase of AIA. Third, proportions of dorsal root ganglion cells express all IL-17 receptor subtypes, and all IL-17 subtypes except IL-17E can increase TTX-resistant Na^+^ currents in isolated DRG neurones. These data will be discussed with a focus on the role of IL-17A in inflammation and on the significance of IL-17 for inflammation-induced pain.

The indistinguishable time course and severity of AIA in WT and IL-17AKO mice does not support a critical role of IL-17A in the pathogenesis of AIA. The preserved anti-inflammatory effect of sympathectomy in IL-17AKO mice supports this conclusion. Previously we showed that sympathectomy in WT mice significantly reduced AIA severity, and this marked anti-inflammatory effect was particularly associated with a strong reduction of the release of IL-17A from antigen-restimulated lymphocytes^28^. Because in WT mice the reduced release of IL-17A from lymphocytes was strongly correlated with the reduction of inflammation of the knee (r = 0.655, p = 0.002, n = 20) whereas the release of other cytokines (IL-2, IL-4, interferon-γ [IFN-γ]) and the concentration of immunoglobulins (IgG2a, IgG2b) were only slightly altered^28^ we hypothesized that the reduction of IL-17A may be a key mechanism of the anti-inflammatory effect of sympathectomy. The preserved significant anti-inflammatory effect of sympathectomy in IL-17AKO mice does not confirm this hypothesis. Thus the development of AIA as well as the reduction of inflammation by sympathectomy is unaltered by the IL-17A knockout.

One possibility is that in IL-17AKO mice the effects of IL-17A are compensated by other IL-17 family members. We found an expression of other members of the IL-17 family in the knee joint and the spleen, and a reduction of mRNA levels of IL-17B, IL-17D and IL-17E after sympathectomy in comparison to IL-17AKO AIA control mice. In splenic lymphocytes of IL-17AKO mice we checked whether there is a release of IL-17F because IL-17A and IL-17F are closely related (for review see Iwakura *et al*.^31^. These IL-17 isoforms share 50% amino acid sequence identity, are secreted as homo- and heterodimers, and the heterodimer of IL-17RA and IL-17RC is the receptor of both cytokines. IL-17A and IL-17F are produced by the same cells (CD4^+^, CD8^+^, γ∂T, NKT cells) and share some of their targets (e.g. fibroblasts and synoviocytes). However, the release of IL-17F from lymphocytes was not reduced after sympathectomy, and thus the significant reduction of the release of IL-17A by sympathectomy in WT mice was not compensated by a reduction of the release of IL-17F in IL-17AKO mice. The redundancy of IL-17 isoforms in inflammation pathogenesis needs to be further studied. E.g. in the CIA model, neutralisation of IL-17B suppressed the progression of arthritis^32^.

However, it is difficult to identify the mechanisms of the pronounced anti-inflammatory effect of sympathectomy in IL-17AKO mice. We found a slight increase of the percentage of T helper 1 (T-bet^+^) and T regulatory (FoxP3^+^) cells and small increases of interferon-γ and IL-10 respectively. These effects may contribute to the anti-inflammatory effect of sympathectomy because interferon-γ inhibits the severity of AIA^33^. Additionally, sympathectomised IL-17AKO mice showed a reduction of the release of CGRP. This neuropeptide is released from sensory neurones, and it was found to be significantly increased in the serum, knee and spleen during the initial swelling phase of AIA^30^. Thus the reduction of CGRP release may have contributed to the anti-inflammatory effect of sympathectomy in IL-17AKO mice.

While IL-17A does not play a major role in the inflammatory process of AIA, it nevertheless significantly contributes to inflammatory pain in AIA. Although IL-17AKO mice showed guarding of the leg (which may indicate pain as well as mechanical factors due to swelling and destruction), they did not exhibit the secondary mechanical hyperalgesia at the paw which is consistently observed in WT mice^21, 28, 30^. Secondary mechanical hyperalgesia remote from the site of inflammation indicates pronounced sensitization of the nociceptive system induced by arthritis in the knee^34^. In WT mice sympathectomy reduced both the severity of AIA and secondary mechanical hyperalgesia^28^. In IL-17AKO mice sympathectomy did not significantly modify the mechanical pain threshold at the ipsilateral paw, plausibly because mechanical hyperalgesia was already abolished by the lack of IL-17A. Thermal secondary hyperalgesia was not dependent on IL-17A (see below). Collectively, these data support previous behavioural/systemic studies of several groups including ours, which show the potency of IL-17 to induce pain^21–25^. Importantly, however, the present data reveal for the first time that IL-17A contributes to arthritic pain even if it is not involved in the pathogenesis of inflammation.

The present study extends previous knowledge of putative cellular nociceptive functions of IL-17 in sensory (DRG) neurones^21, 25^ (i) by demonstrating the expression of different IL-17 receptor subtypes in the DRG, (ii) by showing the effects of IL-17A-F on TTX-R and TTX-S Na^+^ currents in DRG neurones, and (iii) by demonstrating the facilitation of the release of CGRP from DRG neurones by IL-17A. These data clearly show that neurones are targets of IL-17 cytokines.

PCR as well as immunohistochemistry showed the expression of IL-17RA, IL-17RB, IL-17RC, IL-17RD, IL-17RE in DRG neurones, and IL-17RA and IL-17RC were also localised in satellite cells. It is likely that some of the receptor subtypes are coexpressed because different IL-17 receptor subtypes form dimers either as homodimers, e.g. of IL-17RB (activated by IL-17B), IL-17RD (activated by an unknown ligand), IL-17RE (activated by IL-17C), or as heterodimers of IL-17RA and IL-17RC (activated by IL-17A and IL-17F), IL-17RA and IL-17RB (activated by IL-17E), and IL-17RA and IL-17RD (activated by an unknown ligand)^2^. Thus sensory neurones may be activated by diseases characterized by the involvement of different IL-17 isoforms. Except IL-17E (also called IL-25) all IL-17 family members can induce the expression of pro-inflammatory cytokines such as IL-1β, IL-6 and TNF, and all of them are involved in various inflammatory disorders^31, 35^. IL-17E is produced by immune, epithelial, and endothelial cells, and promotes the production of Th2-type cytokines (IL-4, IL-5 and IL-13). It contributes to host defence against parasitic infections and to allergic airway inflammation^36–38^.

Patch clamp recordings had the aim to investigate the effects of different IL-17 family members on DRG neurones. As readout parameters for IL-17 effects we studied changes of TTX-S and TTX-R Na^+^ currents because all nociceptive neurones express voltage-gated Na^+^ channels such as Na_v_1.7 (TTX-S), Na_v_1.8, and Na_v_1.9 (both TTX-R) whereas only subsets of neurones respond to stimuli such as capsaicin and others^39–42^. The recording of voltage-gated Na^+^ currents is justified because IL-17A increases the excitability of small- to medium-sized DRG neurones^21^. TTX-R Na^+^ currents are also increased by other cytokines such as TNFα^43^. To our knowledge the effect of IL-17 on Na^+^ currents was not previously studied. Na_v_1.7, expressed throughout the sensory neurone, is involved in the initial depolarization of the neurone^39^. Na_v_1.8 is opened at more depolarized membrane potentials and carries most of the Na^+^ influx in DRG neurones during the action potential. It is thought to be involved in the generation of action potentials at the sensory endings^39^ and in the excitation of neurones by noxious mechanical stimuli^41, 44^. The slowly activating and inactivating Na_v_1.9 contributes to the regulation of excitability^39^. In the presence of TTX IL-17A enhanced I_max_ of TTX-R currents and caused a shift of activation (G/G_max_) and inactivation (I/I_max_) towards hyperpolarization. Such sensitizing effects are typical for inflammatory mediators such as PGE_2_ and TNFα, and the increase of these currents is thought to be involved in inflammation-evoked hyperalgesia^43, 45–47^. IL-17B, IL-17C, and IL-17D (and IL-17F) caused similar effects in proportions of neurones tested. Remarkably, only one neurone showed an effect of IL-17E. This is interesting because IL-17E has a different profile than the other IL-17 members, more involved in allergy (which is usually not painful) than in other inflammatory disorders (see above). We could not identify an effect of the IL-17 isoforms on TTX-S Na^+^ currents. Similar as the control neurones the neurones tested with IL-17 isoforms showed some run down during the recordings.

Although different IL-17 subtypes increased the maximal TTX-R Na^+^ currents, the lack of IL-17A was sufficient to eliminate secondary mechanical hyperalgesia in AIA indicating that IL-17A is particularly important in AIA-induced mechanical hyperalgesia. A reduction of mechanical hyperalgesia, albeit associated with an attenuation of inflammation was also observed in humans who received a fully human antibody against IL-17A^48^. Thermal secondary hyperalgesia was not affected, similar as in zymosan-evoked paw inflammation^25^. However, the selective effect on mechanical hyperalgesia is probably not totally explained by the effect on voltage-gated Na^+^ channels. IL-17A particularly upregulates the TRPV4 but not TRPV1 receptor in DRG neurones^25^. Whereas TRPV1 is essential for the development of inflammation-evoked thermal hyperalgesia^42^, TRPV4 is not only gated by temperature in the innocuous range with a maximum at 37 °C, it is also considered a candidate molecule for the transduction of noxious mechanical stimuli, at least under inflammatory conditions^49–51^. Here we found that IL-17A enhanced the KCl-evoked release of CGRP from isolated DRG neurones which indicates the activation of nociceptive neurones. The activation of TRPV4 by specific agonists can release CGRP from sensory neurones *in vivo* ^52, 53^.

In sum, the present study shows that IL-17A is involved in inflammation-evoked mechanical hyperalgesia in AIA even though the inflammatory process is not dependent on IL-17A. Thus the beneficial clinical effect of IL-17A neutralisation in some forms of arthritis is likely to result not only from the interference with the inflammatory process but also from the interference with processes of nociception. It may be worth to explore the role of IL-17 signalling in cases of RA in which neutralisation of TNF or IL-6 significantly reduces the inflammatory process but not the pain. Such cases are quite common^54, 55^.

## Methods

### Animals

We used C57BL/6 J male/female wild-type (WT) mice and male/female IL-17A knock-out (KO) mice with C57BL/6 J background (generated by Yoichiro Iwakura)^56^ for AIA experiments. Briefly, for the generation of IL-17AKO mice exon 1 and 2 of the IL-17A gene were replaced with the EGFP (enhanced green fluorescent protein) gene and the neomycin resistance (NeoR) gene flanked by LoxP sequences in the wild-type allele. NeoR was deleted after Cre-Lox recombination^56^. Patch clamp recordings were performed from DRG neurones prepared from WT mice as well as from mice lacking the functional Scn10a (Na_V_1.8^−/−^) gene^41^, kindly provided by Prof. S.H. Heinemann (Center for Molecular Biomedicine, Department of Biophysics, University Hospital Jena). At the end of the experiments mice were killed by cervical dislocation under deep isoflurane (5.0%) anaesthesia.

All mice were bred under the same pathogen-free conditions by the Animal Facility of the University Hospital Jena. All experiments on animals at the University of Jena were performed according to the Tierschutzgesetz der Bundesrepublik Deutschland and approved by the Thüringer Landesamt für Lebensmittelsicherheit und Verbraucherschutz, Abteilung Gesundheitlicher Verbraucherschutz, Veterinärwesen, Pharmazie. The animals were treated in accordance with the declaration of Helsinki and the guiding principles in the care and use of animals. Data sampling, evaluation, and presentation complied with the ARRIVE guidelines.

### Arthritis induction, assessment of AIA and sympathectomy

Mice were immunised at 21 and 14 days before AIA induction with subcutaneous injection of 100 µg of methylated bovine serum albumin (mBSA), the antigen (Sigma-Aldrich, Taufkirchen, Germany), emulgated with 50 µl of complete Freund’s adjuvant (CFA; Sigma-Aldrich), supplemented to 2 mg/ml Mycobacterium tuberculosis, strain H37Ra (Difco, Detroit, USA). Additionally, 5 × 10^8^ heat-inactivated Bordetella pertussis germs (Chiron-Behring, Marburg, Germany) were applied intraperitoneally. For induction of monoarticular AIA 100 µg mBSA in 25 µl 0.9% NaCl was injected into the right knee joint cavity on day 0 under short isoflurane (2.5%) anaesthesia.

Knee swelling was assessed by measuring the medio-lateral joint diameter using an Oditest vernier caliper (Kroeplin, Schlüchtern, Germany). For histopathology, knee joints were removed, fixed in 4.5% formalin, decalcified in EDTA, embedded in paraffin and cut into 3 µm frontal sections which were stained with hematoxylin and eosin (H&E). The pathologist who scored arthritis was unaware of the experimental groups, and arthritis was assessed as previously established^57^. Briefly, signs of acute inflammation (infiltration of the synovial membrane by granulocytes and exudation of granulocytes into the joint space), and chronic inflammation (hyperplasia of synovial lining cells, infiltration of the synovial membrane by mononuclear cells, fibrosis of the synovial membrane and the periarticular tissue) were scored: 0: no, 1: mild, 2: moderate, 3: severe changes (+1 if fibrin exudation in the joint space). Cartilage surface defects with cell necrosis were scored: 0: no damage, 1: < 5%, 2: 5–10%, 3: 11–50%, and 4: > 50% of the cartilage surface affected. Damage to bone was evaluated: 0: no, 1: mild, 2: medium, 3: severe (extensive area of deep invasive destruction of bone). Scoring was graded in 0.5 steps.

For chemical sympathectomy 150 mg/kg 6-hydroxydopamine (6-OHDA; Sigma-Aldrich) in 0.1% ascorbic acid was injected intra-peritoneally on three consecutive days at the time of AIA induction (day −1 until day 1)^28^.

### Pain-related behaviour

Secondary mechanical and thermal hyperalgesia at the hindpaws were assessed as an indicator of pain. After accommodation to the device the mechanical pain threshold was assessed with a dynamic plantar aesthesiometer (Ugo Basile, Comerio, Italy) which applied increasing pressure at 1 g/s to the paw (cutoff at 10 g). The latency of the elicited leg withdrawal which reflects the mechanical threshold was averaged from up to three consecutive stimuli. Two testing’s before AIA induction defined the baseline (BL). Thermal hypersensitivity was assessed using the Hargreaves plantar test (Ugo Basile)^58^. Three consecutive standardized heat stimuli were applied to the paw to evaluate the mean withdrawal latency (cutoff at 20 s). Data are given in gram or seconds as alteration related to BL (d(x)–BL). To quantify the magnitude of hyperalgesia we calculated the area under the curve (AUC) for the values of the right (ipsilateral side of inflammation) and left (contralateral side) paw^59^.

### Cytokine analysis

We used single cell suspensions from spleens, removed at day 2 of AIA. Because lymphocytes show little basal release of cytokines *in vitro*, lymphocytes (10^6^ cells/ml) were cultured for 42 hours with 25 µg/ml mBSA for antigen-specific restimulation. Cytokines from cell culture, except IL-17F, were measured by Luminex Multiplex Detection technology using the Milliplex MTH17MAG-47K-09 kit (Millipore, Schwalbach, Germany) according to the manufacturer’s instructions. IL-17F was measured with a Quantikine^®^ ELISA kit (R&D Systems, Wiesbaden, Germany). Cytokines in serum were measured using standard sandwich ELISA procedures^28^. Primary and biotin-labelled secondary antibodies for IL-2 and IL-6 were purchased from BD Biosciences (Heidelberg, Germany) and for IL-1β, interferon-γ (IFN-γ) and tumor necrosis factor (TNF) α from eBioscience (Frankfurt, Germany) respectively. For quantification, recombinant cytokines were used as standard.

### Serum antibody levels

Immunoglobulins (IgG) specific for the antigen mBSA were determined in serum, obtained at day 2 of AIA, by ELISA^28^. IgG levels are illustrated as the value of absorbance representing readings obtained at 492 nm for total IgG and at 405 nm for the IgG subclasses, respectively.

### Flow cytometry

We used single cell suspensions from spleens, removed at day 2 of AIA. After harvest cells were washed once in PBS by centrifugation at 300× g for 6 min at 4 °C. Cells were stained with the surface antibodies (anti-CD4-APC/eFluor780, eBioscience, 1:200 dilution) including blocking reagents (10 µg/ml anti-CD16/CD32 and rat IgG, Dianova, Hamburg, Germany) for 20 min at 4 °C. After another washing step with PBS cells were fixed and permeabilised according to the manufacturer’s protocol (FoxP3/Transcription Factor Staining Buffer Set, eBioscience). Next, antibodies against intranuclear targets (anti-Tbet-PerCP/Cy5.5, eBioscience, 1:80 dilution), anti-GATA3-PE (eBioscience, 1:20 dilution) and anti-FoxP3-eFluor450 (eBioscience, 1:300 dilution) were added and cells were incubated for 30 min at room temperature. Last, cells were washed in 1x permeabilisation buffer and recorded in a BD LSR II flow cytometer (BD Biosciences). Data was analysed using the FlowJo Software (Treestar, Ashland, Oregon, USA). Supplementary Figure S3 displays a representative example of the gating strategy for flow cytometric analysis.

### Polymerase chain reaction (PCR) of IL-17A-F and IL-17 receptors (IL-17R)

Total RNA was obtained from mouse tissues as indicated (DRG, spleen, knee joint) using an RNeasy Plus Mini kit (Qiagen, Hilden, Germany). For reverse transcription we used a RevertAid First Strand cDNA synthesis kit (Thermo Fisher Scientific, Waltham, MA, USA). All messenger RNA templates were transcribed in cDNA using the oligo(dT)_18_ primer. Primers and annealing temperatures were as described^32^ as follows: IL-17A 5′-GCTCCAGAAGGCCCTCAGA-3′ (forward) and 5′-AGCTTTCCCTCCGCATTGA-3′ (reverse) (142 bp) 61 °C, IL-17B 5′-CGGTGCCTATGTTTGGGTTGC-3′(forward) and 5′-GGGTTGGTGGTTGGCTCAGAA-3′ (reverse) (201 bp) 63 °C, IL-17C 5′-CACAGATGAGAACCGCTACCC-3′ (forward) and 5′-GCGGATGAACTCGGTGTGGAA-3′ (reverse) (211 bp) 60 °C, IL-17D 5′-GCTCTACGGGGAGGAGGAC-3′ (forward) and 5′-GATCATGGGGTGGGTTTTG-3′ (reverse) (310 bp) 63 °C, IL-17E 5′-GACCTGTACCACGCTCGATG-3′ (forward) and 5′-CCTGCTCCTTCCCAAAAGTG-3′ (reverse) (420 bp) 60 °C, IL-17F 5′-CAACGCTGCATACAAAAATCA-3′ (forward) and 5′- TTAAGTGAGGCATTGGGAACA-3′ (reverse) (220 bp) 60 °C, IL-17RA 5′-CATCACACTCATCGCCATTC-3′ (forward) and 5′- GCCGAGTAGACGATCCAGAC-3′ (reverse) (186 bp) 60 °C, IL-17RB 5′-CAGCATCCGCTTGTTGAAG-3′ (forward) and 5′-CTGGTCTGGCTTTGGAAGG-3′ (reverse) (111 bp) 59 °C, IL-17RC 5′-GTCAGTCCGTGGGTTCTGC-3′ (forward) and 5′-CTGAGGTCCAGTCAGGTTTTTG-3′ (reverse) (320 bp) 66 °C, IL-17RD 5′-CCCTGTATGTTGCCATTTGC-3′ (forward) and 5′-GACTGCCAGCTTTCACTGC-3′ (reverse) (348 bp) 61 °C, IL-17RE 5′-GCATCCTGGTGTGGAGGTC-3′ (forward) and 5′-CCTTCCCAGAGATCCACGAT-3′ (reverse) (298 bp) 65 °C. For PCR (MyCycler Thermal Cycler, Bio-Rad, Hercules, CA, USA) we used Phire Hot Start II DNA Polymerase (Thermo Fisher Scientific). The Master Mix consisted of 1x PCR buffer, 1 unit Taq DNA polymerase per 50 µl PCR mixture, 0.2 mM dNTP mixture (10 mM dNTP Mix; Thermo Fisher Scientific), and 0.5 µM primer. For amplification, 20–24 µl of Master Mix plus 1–5 µl cDNA template were used. Reaction conditions were as follows: step 1, 1–3 minutes at 98 °C; step 2, 5–15 seconds at 98 °C; step 3, 5–10 seconds at annealing temperatures as indicated before; step 4, 10–20 seconds at 72 °C; and step 5, 1 minute at 72 °C. Steps 2–4 were repeated 30–35 times. PCR mixtures were analysed with 2% Tris–acetate–EDTA agarose gel containing ethidium bromide and a low range DNA ladder (Fermentas, Thermo Fisher Scientific) as a size standard or with Agilent 2200 TapeStation technology according to the manufacturer’s instructions (Agilent, Santa Clara, California, USA).

### Real-time reverse transcriptase (RT) PCR

Knee joints, removed at day 2 of AIA, were snap-frozen without further dissection and manually homogenized by pestle and liquid nitrogen. Cell lysis and total RNA isolation was performed using Trizol reagent (peqGOLD TriFast, PEQLAB, Erlangen, Germany) according to the manufacturer’s instruction, and an ultrasonicator (Covaris, Brighton, UK) at 70 W for 2 min. Probes were stored at -80 °C until usage. The QuantiTect Reverse Transcription Kit (Qiagen) was used for cDNA synthesis. Quantitative RT-PCR was done using the Rotor-Gene 6000 system (Qiagen) and FastStart Universal SYBR Green Master (Roche, Mannheim, Germany). The Master Mix consisted of 6.25 µl SYBR Green, 0.375 µl forward primer, 0.375 µl reverse primer and 5 µl H_2_O in a total volume of 12 µl. For amplification, 12 μl of Master Mix plus 0.5 μl cDNA template were used. Primers were the same as described before, and additionally for glyceraldehyde-3-phosphate dehydrogenase (GAPDH): 5′-CACACCGACCTTCACCATTTT-3′ (forward) and 5′-GAGACAGCCGCATCTTCTTGT-3′ (reverse). Reaction conditions were as follows: step 1, 10 minutes at 95 °C; step 2, 5 seconds at 95 °C; step 3, 20 seconds at 60 °C; step 4, 25 seconds at 72 °C. Steps 2–4 were repeated 40 times. GAPDH served as housekeeping gene and the relative expression value of each gene was normalized to GAPDH for each sample. mRNA level of tissues from sympathectomised IL-17AKO mice were displayed in relation to control IL-17AKO mice.

### Localisation of IL-17 receptors in DRG sections

For immunohistological localisation of the IL-17 receptors ganglia from mice embedded in paraffin were used. The sections were dewaxed and autoclaved for 15 min (120 °C, 1 bar) in 0.1 mol/l citrate buffer (pH 6.0). Chilled and PBS-washed sections were incubated for 30 min in PBS containing Triton-X100 and 2% goat serum or 2% rabbit serum (Dako Glostrup, Denmark), and then incubated overnight at 4 °C with the primary anti-IL17 receptor antibodies diluted in PBS containing 1% Triton X-100 and 1% gelatine from cold water fish skin in a moist chamber. The following antibodies were used: anti-IL-17RA antibody (rabbit polyclonal, # sc-30175), anti-IL-17RB (goat polyclonal, # sc-11754) both diluted 1:100 (Santa Cruz Biotechnology, Santa Cruz, Califonia, USA); anti-IL-17RC (# orb5534), anti-IL-17RD (# orb5539) and anti-IL-17RE (# orb 179050) all rabbit polyclonal antibodies diluted 1:50 (Biorbyt, Cambright, UK). After incubation the PBS-washed sections were incubated for 2 hours with a biotinylated goat anti-rabbit antibody or a biotinylated rabbit anti-goat antibody (1:200; Dako) at 20 °C. After 3 washes with PBS the avidin-biotin peroxidase complex (Vectastatin-Elite ABC Kit, Vector Laboratories, Burlingame, USA) was applied for 40 min. Sections were developed with Jenchrom px blue (MoBiTec, Göttingen, Germany), dehydrated and embedded in Entellan (Merck). Control experiments were carried out with omission of the primary antibodies. Labelling of the different anti-IL-17 receptor antibodies were documented using a light microscope (Axioplan 2, Zeiss, Jena, Germany) coupled to an image analysing system (Axiovision, Zeiss).

### Cell culture of DRG neurones

DRGs from mice were dissected, incubated in 125 U/ml collagenase type II (Paesel and Lorei, Hanau, Germany), diluted in Ham’s F12 (PAA, New Jersey, USA), for 1 hour at 37 °C. After washing ganglia were placed in 10,000 U/ml Trypsin (Sigma), diluted in Dulbecco’s modified Eagle’s medium (DMEM, Gibco, BRL, Eggenstein-Leopoldshafen, Germany) for 11 min at 37 °C. Neurones were separated by gentle agitation and mechanical treatment with a fire polished Pasteur pipette. The cell suspension was centrifuged at 500xg (8 min), finally neurones were resuspended in media consisting of Ham’s F12, 10 ng/ml nerve growth factor (Enzo, Lörrach, Germany), 2 mmol/l glutamine (Sigma), 15% heat inactivated horse serum (PAA), 100 U/ml penicillin, and 100 μg/ml streptomycin (Gibco). The cell suspension was plated in poly-L-lysine- (200 µg/ml; Sigma) coated cell culture dishes and kept in a humidified incubator gassed with 5% CO_2_ and air at 37 °C. After 24 h or 48 h (as indicated) of cell culture the supernatants were removed and cells were fed with fresh media.

### Measurement of CGRP release from DRG neurones

The release of CGRP from DRG neurone cell cultures was induced as previously described^29^. Basal CGRP production was measured in supernatants after culture for 20 min. For stimulation cells were fed with fresh media and we applied either 50 mmol/l KCl alone for 20 min, or KCl with IL-17A (for concentrations see Fig. 8g). After the final removal of the supernatants DRG neurones were harvested for protein assay using RIPA lysis buffer. Probes were stored at -20 °C until usage.

### CGRP measurement and protein assay in tissue

CGRP was quantified in supernatants from cultured DRG neurones, in serum and tissue extracts from knee joints as described previously^30^. Briefly, the tissues were snap-frozen without further dissection, and extracts were prepared in an extraction buffer (pH 3.4) containing protease inhibitors using an ultrasonicator (Covaris) for lysis after manual tissue homogenization by pestle and liquid nitrogen. Total protein concentration in tissue extracts and from DRG cell cultures was determined with a BCA protein assay (Thermo Fisher Scientific) according to the manufacturer’s instructions. For CGRP measurement we used enzyme immunoassay kits (for serum and tissue extracts: Cusabio, Wuhan, China; for cell culture supernatants: Bertin pharma, Montigny le Bretonneux, France) according to the manufacturer’s instructions.

### Whole-cell patch clamp recordings from cultured DRG neurones

Standard whole-cell patch clamp experiments from small and medium sized (≤35 µm diameter) DRG neurones were performed at room temperature using an EPC-10 USB-double amplifier in combination with PATCHMASTER software (HEKA Electronics, Lambrecht, Germany). For recording of TTX-R Na^+^ currents the cells within 8 h after plating were continuously superfused with standard bath solution (in mM): 35 NaCl, 78 choline-Cl, 5 KCl, 30 TEA-Cl, 1.8 CaCl_2_, 1 MgCl_2_, 0.1 CdCl_2_, 10 glucose, 10 HEPES, TTX 500 nM, 5 4-aminopyridine (4-AP). TEA and 4-AP were added to block K^+^ currents and CdCl_2_ was added to block Ca^2+^ currents. The pH was set to 7.4 with HCl, the osmolarity was adjusted to 300 mOsm/kg with glucose. For recording of TTX-S Na^+^ currents we used cells from Na_V_1.8^-/-^ mice because TTX-R Na_v_1.8 currents can contaminate the recording of TTX-S currents. The standard bath solution was the same except that no TTX was added. Testing compounds were IL-17A-F, final bath concentrations 50 ng/ml. Patch pipettes were made by two step vertical PC-10 puller (Narishige, Tokyo, Japan) from borosilicate glass (Kimble Chase Gerresheimer, Santiago de Querétaro, Mexico) and filled with (internal) solution (in mM): 10 NaCl, 110 CsCl, 20 TEACl, 2.5 MgCl_2_, 5 HEPES, 5 EGTA. The pH was adjusted to 7.0 with CsOH. Osmolarity was adjusted to 300 mOsm/kg with sucrose. Current signals were low-pass filtered at 1.1 kHz (4-pole Bessel) and 20 kHz (Bessel). For presentation, data were then digitalized at 500 Hz. Capacitive and leakage currents were subtracted digitally by the P/4 protocol. The data were analysed using the FITMASTER (HEKA Electronics, Lambrecht, Germany) and Origin 8.1 G (Microcal Software, Northampton, MA) software programs. Current densities were calculated by dividing the peak current (I_peak_) evoked at each membrane potential (V_m_) by the cell capacitance (C_m_). The peak conductance (G) of Na^+^ currents at each potential was calculated from the corresponding peak current by using the equation G = I/(E − E_Rev_) (E_Rev_: reversal potential of Na^+^ current; I: peak current amplitude of Na^+^ current; E: membrane potential).

For determination of the steady-state inactivation a double-pulse protocol was used. Starting again from a holding potential of -80 mV the membrane was first depolarized for 500 ms in 5 mV steps in a range of −75 to + 10 mV, and then the membrane was depolarized to −5 mV for 100 ms.

Normalised peak conductance (G/G_max_) and steady-state inactivation (I/I_max_) were fitted with a Boltzmann function G/G_max_ (I/I_max_) = [1 + exp((V_½_ − V_m_)/k)]^−1^ where V_½_ is the membrane potential generating half maximal activation or inactivation, V_m_ is the membrane potentials (or prepulse potential in steady-state inactivation experiments), and k is the slope of the function. For the display of I/V curves the average peak currents at each voltage test were used.

In some neurones we tested the excitability using current-clamp recordings. In these experiments the bath was perfused with HEPES solution (control; 118 mM NaCl, 5 mM KCl, 2 mM CaCl_2_, 2 mM MgCl_2_, 10 mM glucose, and 10 mM HEPES, pH 7.4). Test compounds were added with an application system. The recording pipettes contained 140 mM KCl, 10 mM NaCl, 1 mM MgCl_2_, 0.5 mM CaCl_2_, 2 mM Na_2_-ATP, 5 mM EGTA, 10 mM HEPES, and 10 mM sucrose, pH 7.2. We included only neurones with a membrane potential less than −45 mV. To assess neuronal excitability, APs were elicited by current injection through the recording pipette. At the resting potential, current was applied at amplitudes from 0 pA in 25 pA steps (pulse duration 5 msec, interpulse interval 2 seconds) until an AP with the typical overshoot was elicited. This protocol was repeated every 2 minutes before application of IL-17A, and within 3–7 minutes after application of IL-17A to the bath. In addition, ramp current (0 pA to 3x threshold current) was applied for 500 ms, and the latency of the first AP and the numbers of elicited APs were measured before and during IL-17A application.

### Statistical analysis

All data are expressed as means ± SEM unless otherwise stated. Differences between groups were calculated using the two-tailed Student’s t-test for unpaired and normally distributed values (for testing for normality we used the Kolmogorov-Smirnov test). Pain thresholds against baseline values were analysed using the Wilcoxon’s matched-pairs signed-rank test, and Bonferroni correction was used for multiple comparisons. For statistical comparison of current densities before and after treatment the maximal negative peak currents were taken from each neurone irrespective of shifts of maximum currents with respect to voltage, and the paired t-test was used (after testing for normality using the Shapiro-Wilk test). Statistical significance was calculated with SPSS (v.16.0, Chicago, USA) and Origin 8.1 G (Microcal Software, Northampton, MA) software programs, and accepted at p < 0.05.

### Data availability

All data generated or analysed during this study are included in this published article (and its supplementary information files).

## Electronic supplementary material

Supplementary Figures S1-S3
