# Supplementary material for: Interleukin-17A is involved in mechanical hyperalgesia but not in the severity of murine antigen-induced arthritis
Source: Sci Rep. 2017 Sep 4;7:10334. doi: 10.1038/s41598-017-10509-5 (PMC5583382; doi:10.1038/s41598-017-10509-5)
Supplement: Supplementary file 1 — Supplementary Figures S1-S3 [file 41598_2017_10509_MOESM1_ESM.pdf]

Interleukin-17A is involved in mechanical hyperalgesia but not in the severity of murine antigen-induced arthritis

Matthias Ebbinghaus, Gabriel Natura, Gisela Segond von Banchet, Susanne Hensellek (†), Martin Böttcher, Birgit Hoffmann, Firas Subhi Salah, Mieczyslaw Gajda,Thomas Kamradt, Hans-Georg Schaible

Supplementary Figure S1

In parts adapted from published data with permission: Ebbinghaus, M., Gajda, M., Boettger, M.K., Schaible, H.G. & Bräuer, R. The anti-inflammatory effects of sympathectomy in murine antigen-induced arthritis are associated with a reduction of Th1 and Th17 responses. *Ann. Rheum. Dis.* **71,** 253-261 (2012).

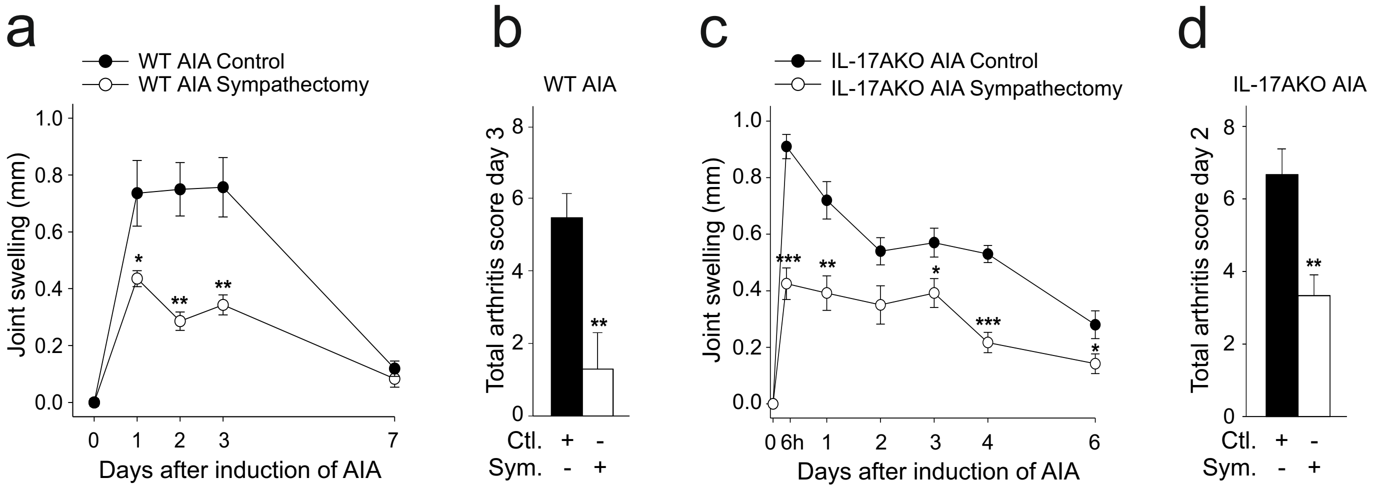

Comparison of joint swelling and histopathological examination between C57BL/6 wild type (WT) mice (adapted from Ebbinghaus, M. *et al. Ann. Rheum. Dis.* **71,** 253-261 (2012)) and IL-17AKO mice in antigen-induced arthritis (AIA) after sympathectomy. (a) Sympathectomy significantly (d1 $p=0.041$, d2 $p=0.002$, d3 $p=0.006$) reduced joint swelling in WT mice in acute AIA as shown before (n = 8 per group). (b) Histological examination of the inflamed knee joints from WT mice at day 3 of AIA showed significant ($p = 0.008$) differences in total arthritis score between sympathectomised mice and AIA controls (n = 8 per group). (c) In IL-17AKO mice sympathectomy reduced joint swelling significantly (6h $p<0.001$, d1 $p=0.005$, d3 $p=0.037$, d4 $p<0.001$, d6 $p=0.043$) in a comparable manner like in WT mice (n = 6 per group). (d) Also the total arthritis score was significantly ($p = 0.005$) reduced in IL-17AKO mice by sympathectomy (n = 6 per group). *$p<0.05$, **$p<0.01$ *Ctl.* AIA Control, *Sym.* AIA Sympathectomy

Interleukin-17A is involved in mechanical hyperalgesia but not in the severity of murine antigen-induced arthritis

Matthias Ebbinghaus, Gabriel Natura, Gisela Segond von Banchet, Susanne Hensellek (†), Martin Böttcher, Birgit Hoffmann, Firas Subhi Salah, Mieczyslaw Gajda,Thomas Kamradt, Hans-Georg Schaible

Supplementary Figure S2

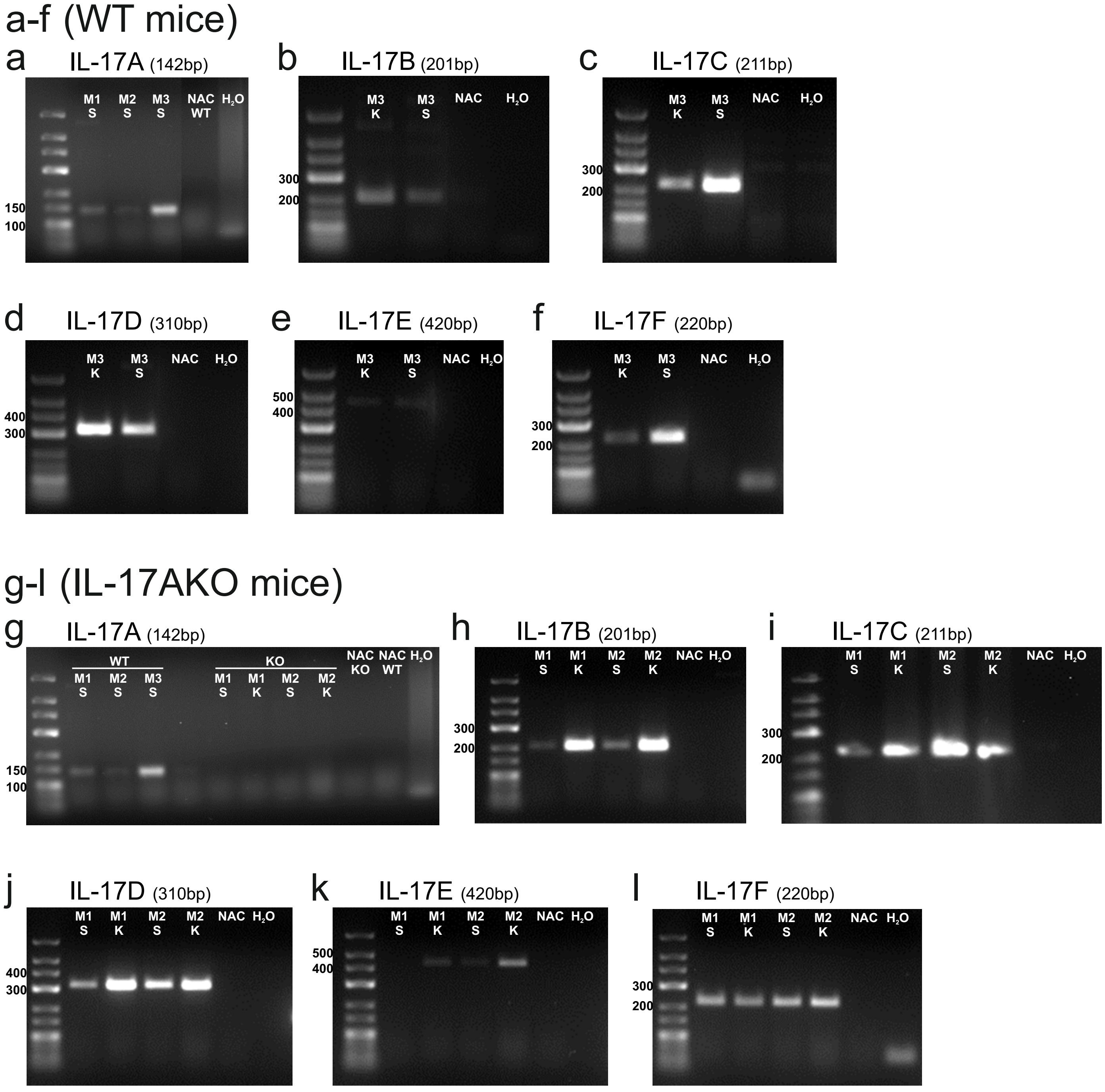

Representative agarose gel images of PCR of IL-17 cytokine family members in spleens and knee joint tissues of immunized wild type (WT) mice and immunized IL-17AKO mice.
(a-f) Individual WT mice show the expression of all IL-17 cytokine family members in various intensities. (g-l) IL-17AKO mice also show all IL-17 cytokine family members except IL-17A. DNA ladder with base pair indication at the left of every single image. M1-M3, mouse 1-3; S, spleen; K, knee joint; NAC, no amplification control; H20, water control; bp, base pairs

Interleukin-17A is involved in mechanical hyperalgesia but not in the severity of murine antigen-induced arthritis

Matthias Ebbinghaus, Gabriel Natura, Gisela Segond von Banchet, Susanne Hensellek (†), Martin Böttcher, Birgit Hoffmann, Firas Subhi Salah, Mieczyslaw Gajda,Thomas Kamradt, Hans-Georg Schaible

Supplementary Figure S3

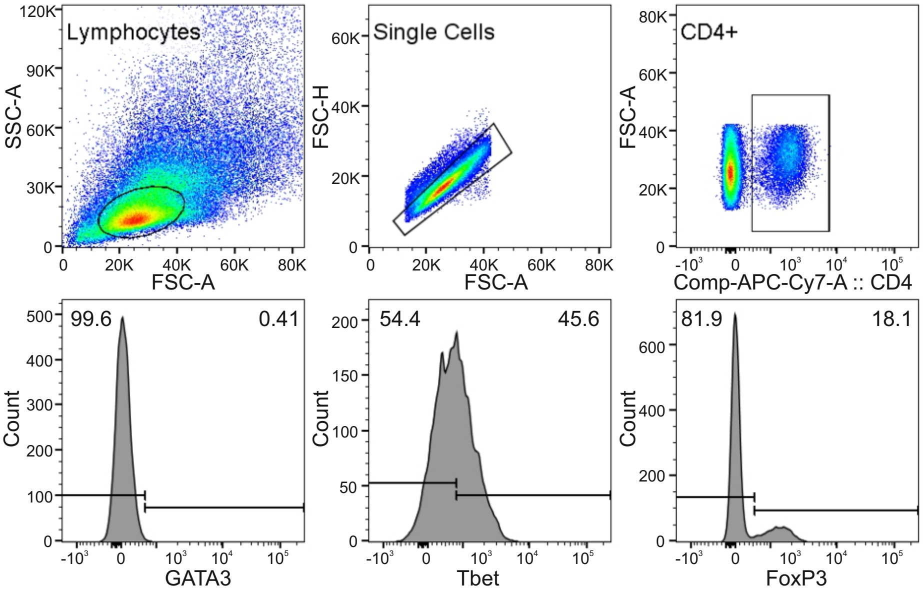

Representative example of the gating strategy for flow cytometric analysis. Cells were stained as detailed in materials and methods. Single lymphocytes were gated based on their forward (FCS) and sideward (SSC) scatter properties. CD4+ lymphocytes were further analyzed for their expression level of the transcription factors T-bet, GATA3 and FoxP3.
